# Catheter-Based Fetal Cardiac Interventions

**DOI:** 10.3390/jcdd11060167

**Published:** 2024-05-29

**Authors:** Betul Yilmaz Furtun, Shaine Alaine Morris

**Affiliations:** Texas Children’s Hospital, Baylor College of Medicine, 6651 Main Street, Suite E1920, Houston, TX 77030, USA

**Keywords:** fetal cardiac intervention, critical congenital heart disease, catheter-based intervention

## Abstract

Fetal cardiac intervention (FCI) is an emerging and rapidly advancing group of interventions designed to improve outcomes for fetuses with cardiovascular disease. Currently, FCI is comprised of pharmacologic therapies (e.g., trans-placental antiarrhythmics for fetal arrhythmia), open surgical procedures (e.g., surgical resection of pericardial teratoma), and catheter-based procedures (e.g., fetal aortic valvuloplasty for aortic stenosis). This review focuses on the rationale, criteria for inclusion, technical details, and current outcomes of the three most frequently performed catheter-based FCI procedures: (1) aortic valvuloplasty for critical aortic stenosis (AS) associated with evolving hypoplastic left heart syndrome (HLHS), (2) atrial septal intervention for HLHS with severely restrictive or intact atrial septum (R/IAS), and (3) pulmonary valvuloplasty for pulmonary atresia with intact ventricular septum (PA/IVS).

## 1. Introduction

FCI describes a group of interventions designed to improve fetal cardiac status in fetuses with cardiovascular disease. The principal objectives of FCI are to enhance fetal hemodynamics and to facilitate cardiac remodeling to potentially alter the natural disease progression in utero, ultimately aiming to improve postnatal outcomes while minimizing maternal complications [1,2,3,4,5].

Currently, there are three main categories of FCI: pharmacologic, open surgery, and instrumental or closed procedures. Pharmacologic therapies encompass the administration of medication to the fetus, most commonly transplacentally (maternally). Examples of this are the administration of anti-arrhythmic medications for fetal arrhythmia, indomethacin for Ebstein anomaly, and oxygen therapy for fetuses with underdeveloped left-sided structures [6,7,8,9,10,11,12]. Open surgical FCI is rarely performed but has been successfully performed for fetal pericardial teratomas when there is risk of fetal demise [13,14]. Catheter and/or needle-based procedures include simpler interventions like pericardiocentesis as well as more complex interventions such as atrial septal dilation and stent placement in HLHS with severely restrictive or intact atrial septum (R-IAS) and valvuloplasty in critical AS and pulmonary valve stenosis [1,2,3,13,15,16,17,18].

Fetal non-cardiac surgery was first performed in humans in 1980s. In 1982, Harrison et al. published a communication in the New England Journal of Medicine, contending that the correction of specific fetal structural abnormalities might facilitate normal fetal progression [1]. During that era, a select group of abnormalities that were found to be amendable to potential improvement by fetal treatment included non-cardiac pathologies like obstructive uropathy leading to renal insufficiency, severe congenital diaphragmatic hernia leading to lung hypoplasia, severe congenital cystic adenomatoid malformation of the lung, and sacrococcygeal teratoma [15,19,20,21,22]. In the ensuing years, primarily owing to advancements in maternal–fetal medicine, surgical techniques, and fetal imaging, many initial technical obstacles have been overcome, a deeper understanding of the natural progression of various fetal disorders has been gained, and the scope of fetal therapy has expanded to encompass fetal cardiac intervention (FCI) [1,2,3].

Research utilizing experimental animal models commenced in the 1980s in hopes of developing intrauterine repair for congenital heart disease [23]. Despite promising initial findings, the open surgery ultimately resulted in fetal hypoxia and mortality due to post bypass placental dysfunction [24]. Consequently, the pursuit of open fetal cardiac surgery was largely discontinued.

The first documentation of human fetal cardiac therapy was reported in 1975 and involved maternal–fetal transplacental administration of a beta blocker to manage fetal ventricular tachyarrhythmia [25]. A decade later, in 1986, the first instance of an open in utero cardiac procedure was reported with pacemaker placement to address complete heart block [26]. The notion of performing catheter-based balloon valvuloplasty in fetuses with stenotic heart valves followed the successful debut of neonatal balloon valvuloplasty in the 1980s, with the first reported case performed in a fetus with critical aortic stenosis (AS) in 1991 [4].

Since that time, catheter-based FCIs have increased across the world [27]. Catheter-based FCI currently is only applicable to certain congenital heart defects (CHDs), particularly those that worsen during mid to late gestation or pose a risk of significant hemodynamic instability, necessitating urgent intervention at birth [1,2,3]. This review will primarily concentrate on the three most commonly performed catheter-based FCIs: balloon aortic valvuloplasty for critical AS with evolving HLHS, atrial septoplasty/stent placement for HLHS with R/IAS, and pulmonary valvuloplasty for pulmonary atresia with intact ventricular septum (PA/IVS). In cases of severe AS and PA/IVS, FCI aims to alter the natural disease trajectory, potentially leading to postnatal biventricular circulation. Despite an increasing number of patients achieving biventricular circulation, careful patient selection and postnatal management strategy are pivotal for ultimate success. Conversely, in HLHS with R/IAS, FCI aims to enhance both prenatal hemodynamics and prevent in utero lung changes to improve postnatal survival, although it does not aim to achieve biventricular circulation, as this is typically not a possibility.

Currently all catheter-based FCIs are conducted via a percutaneous trans-ventricular or atrial approach. In 2013, Edwards and his co-workers successfully performed percutaneous transhepatic cardiac catheterization in a fetal sheep model [28]. They demonstrated that it is possible to navigate the catheter into all four chambers of the heart, accessing the left side chambers through the foramen ovale while simultaneously advancing the catheter into the right ventricle and out through the pulmonary artery into the branch pulmonary arteries or via the patent ductus into the descending aorta. Sham ballooning of both the atrial septum and the pulmonary valve was performed without complications [29]. This technique may offer advantages over the trans-ventricular or atrial approach once further studies aimed at its translation to the human fetus have been completed, although to date we are not aware of this being performed in a human.

## 2. Fetal Aortic Valvuloplasty for Severe Aortic Valve Stenosis (FAV)

HLHS is a congenital heart lesion characterized by significant underdevelopment of left heart structures incapable of supporting systemic circulation [11,12,30,31]. Those having HLHS at birth may present in one of three ways prenatally. The first is when the ventricle is hypoplastic or absent throughout gestation, presumably due to failure of the LV to properly develop embryologically and is most often associated with mitral and aortic atresia. The second is when severe aortic stenosis develops during gestation, resulting in initial left ventricular dilation followed by left ventricular growth arrest and a globular dysfunctional ventricle [32,33]. The third is thought to be due to left-sided inflow obstruction and is when there is severe aortic arch hypoplasia typically seen with mitral and aortic annular hypoplasia but without true valvar stenosis and a narrow but apex-forming left ventricle [34]. This last group actually rarely develops true HLHS and most often can undergo biventricular repair [34,35]. The second group, however, is at high risk of developing HLHS, especially if the AS develops before 30 weeks gestation. This group is the target for fetal aortic valvuloplasty.

**Figure 1:**
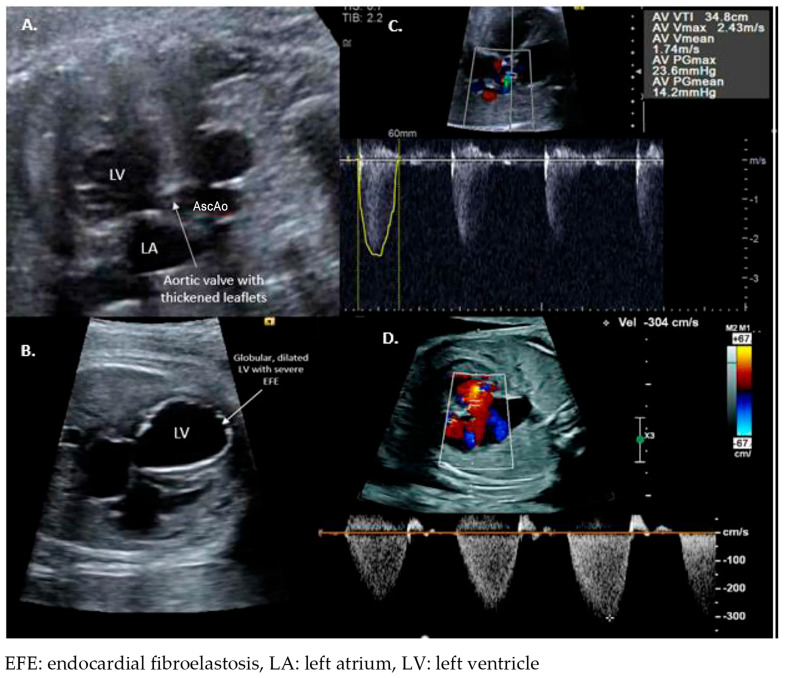
Fetus with critical aortic stenosis. Fetal echocardiogram performed at 29 wks GA. (**A**) Left ventricular outflow view demonstrating thickened aortic valve leaflets. Annulus measures mildly hypoplastic with a z-score of −2.4. (**B**) Four-chamber view demonstrating severely dilated, globular left ventricle with echobright endocardium consistent with endocardial fibroelastosis. (**C**) Doppler interrogation of the aortic outflow tract with a peak velocity of ~2.4 meters per second (m/sec) in the setting of severe left ventricular dysfunction. (**D**). Doppler interrogation of the mitral valve regurgitation predicting left ventricle systolic pressure of ~3 m/sec plus systemic venous pressure.

Initially, mid-gestation severe valvar AS is characterized by decreased mobility of the aortic valve leaflets, a narrow color jet across the left ventricular outflow tract with no or minimal subvalvar aortic obstruction, a normal or dilated left ventricle (LV) with development of endocardial fibroelastosis and systolic dysfunction (Figure 1) [32,36,37,38,39]. These fetuses frequently develop HLHS later in gestation due to LV growth arrest thought to be due to decreased flow across the left heart structures and worsening endomyocardial fibrosis. Characteristics on fetal echocardiogram that predict the progression of mid-gestation fetal AS to HLHS include at least moderate LV dysfunction, either retrograde or bidirectional flow across the transverse aortic arch, left-to-right or bidirectional flow through the patent foramen ovale, and monophasic mitral inflow pattern [33,40,41,42,43]. Progression from a dilated left ventricle with high pressure to a normal sized or hypoplastic left ventricle with no reserve and low ventricular pressure can occur within 1–2 weeks gestation. Fetal aortic valvuloplasty (FAV) has shown promise in preventing mid-gestation critical AS progression to HLHS by relieving obstruction across the aortic valve, improving left ventricular outflow tract flow [4,27,33,44,45,46,47,48]. The primary aim of this procedure is to prevent HLHS progression, ultimately allowing for biventricular circulation at birth.

### 2.1. Patient Selection

The selection criteria for FAV comprise two key components: the high likelihood of developing hypoplastic left heart syndrome (HLHS) without FAV, as previously outlined, and the potential for left ventricular (LV) recovery post-FAV to sustain systemic circulation. Fetal echocardiogram findings that suggest an inability for LV recovery post-intervention or predict single ventricle circulation despite successful FAV include LV hypoplasia (end-diastolic volume or long-axis z-score < −2), mitral valve hypoplasia (z-score < −2), and low LV pressure (estimated LV pressure < 30 mmHg by mitral regurgitation or aortic stenosis jet velocity) [43,44]. Conversely, factors associated with a higher likelihood of LV salvageability to sustain the systemic circulation after birth encompass higher LV pressure (>46 mmHg), larger ascending aorta z-score, and biphasic mitral inflow or indices indicative of normal LV diastolic function. Friedman et al. published a Cartesian and Regression Tree (CART) analysis in 2018 that allows fetal practitioners to enter multiple fetal echocardiographic variables into a model to predict the probability of left ventricular recovery and a successful biventricular outcome [43].

### 2.2. Procedural Technique

FAV is carried out percutaneously under continuous ultrasound guidance once the above criteria are met. The ideal gestational age for treatment is between 18 and 30 weeks’ gestation. Typically, the procedure is performed using epidural anesthesia instead of general anesthesia (GA) to mitigate the risks associated with GA and potential fetal myocardial depression [49]. Conscious sedation may be employed to ensure maternal comfort and reduce movement during the procedure. Intramuscular administration of a combination of medications, including an anesthetic, paralytic agent, and atropine, is administered to the fetus to alleviate pain and facilitate the smooth progression of the procedure.

**Figure 2:**
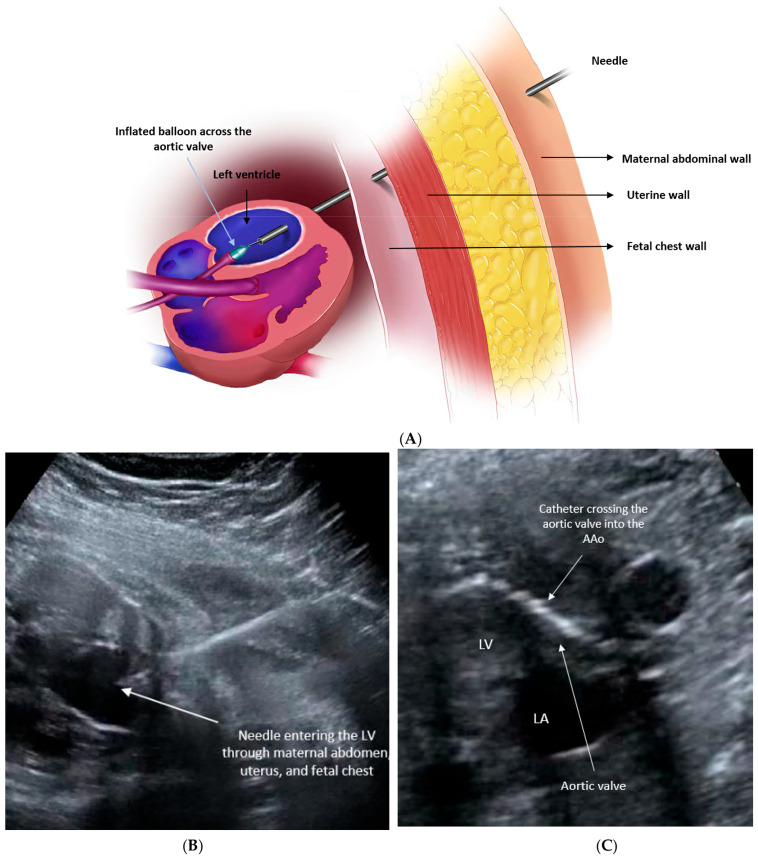
(**A**) Illustration demonstrates needle crossing through the maternal abdominal wall, uterine wall, then fetal chest into the fetal heart, through which the balloon catheter is advanced. (**B**) Image obtained during aortic valvuloplasty for critical aortic stenosis under ultrasound guidance. The needle is seen in the left ventricle apex pointing toward the aortic valve. (**C**) The catheter is seen across the left ventricular outflow tract.

An 18- or 19-gauge needle is then inserted percutaneously into the fetal chest, ideally targeting the left ventricular (LV) apex and directed toward the LV outflow tract. Subsequently, a wire with an attached balloon is threaded through the needle and passed across the narrowed aortic valve. Once in an optimal position, the coronary angioplasty balloon is gently inflated. This inflation enlarges the opening of the aortic valve, facilitating increased blood flow into the left ventricle. Upon completion of the procedure, the balloon is deflated, and the balloon, wire, and needle are removed (Figure 2). The technical success of the procedure is determined by the improvement in forward flow across the aortic valve and/or the development of new aortic regurgitation, which usually is no more than mild and tends to improve or resolve after birth due to valve remodeling [3,41,46].

### 2.3. Procedural Outcomes

Since its initial attempts in the 1990s, procedural outcomes for FAV have demonstrated notable improvement. The outcomes from the International Fetal Cardiac Intervention Registry (IFCIR), which included data from 15 institutions worldwide on 108 fetuses undergoing FAV 2002–2018, reported a technical success rate of 83% with a fetal demise rate of 17% [50]. The largest reported cohort of fetuses undergoing FAV, spanning from March 2000 to December 2017 at Boston Children’s Hospital, encompassed 143 cases [51]. Within this cohort, FAV achieved technical success in 84% of fetuses, representing a significant advancement compared to earlier studies, which reported a success rate of 70–74% [41,47]. Fetal demise occurred in 8% of cases. Analyzing the subset of cases from 2009 onward (n = 71) to reflect current practice, all outcomes demonstrated improvement, with technical success reaching 94% and fetal demise reducing to 4% in the latest era. Apart from fetal death, fetal complications such as bradycardia, ventricular dysfunction, and hemopericardium are common but reversible, often responsive to interventions like intracardiac epinephrine administration and pericardiocentesis [27,45,48,51].

### 2.4. Post-Procedural Outcomes

Prosnitz et al. reported acute hemodynamic changes in fetal hearts post-FAV, evidenced by improved parameters of left ventricular diastolic function and modest enhancement in left ventricular systolic function on immediate post-procedural fetal echocardiograms [52]. The development of prograde aortic arch flow and bidirectional flow across the atrial septum predicting postnatal biventricular circulation are reported to occur in approximately 50% of fetuses. Additionally, improved growth of left-sided structures has been documented in fetuses undergoing successful FAV compared to those without fetal cardiac intervention or those with unsuccessful FAV [27,41,43,48].

### 2.5. Postnatal Outcomes

Early findings suggest that FAV may enhance the likelihood of achieving a biventricular repair, but this intervention carries the risk of fetal mortality, and its impact on overall mortality rates and outcomes remains uncertain with mixed results. Consequently, the potential benefits of FAV must be carefully weighed against the known risk of fetal demise, as well as the possibilities of a technically unsuccessful procedure or postnatal single ventricle (SV) circulation despite successful FAV.

In a study conducted by Freud et al., which examined the outcomes of the initial 100 fetuses undergoing FAV for severe mid-gestation aortic stenosis with evolving HLHS at Boston, with a median follow-up of 5.4 years, it was found that 38 out of the 88 infants born alive (39%) achieved a biventricular circulation, either directly from birth or following initial univentricular palliation [48].

While long-term outcomes are still undetermined, it has been observed that FAV may confer medium-term survival benefits (between ages 3 to 6 years) compared to expectant fetal management, provided that patient selection is appropriate, as it has been identified as a critical factor for obtaining survival benefits. A secondary decision analysis using both the Boston Children’s cohort (n = 143) and data from the Pediatric Heart Network Single Ventricle Reconstruction trial was conducted [51]. Using these findings, the authors developed a decision model to estimate the probability of transplant-free survival from fetal diagnosis to a specific age. This decision model was designed to address the uncertainty between expectant management and attempted FAV, identifying key parameters that influence potential benefits. The model projected overlapping probabilities of transplant-free survival to age 6 at 75% (95% CI 67–82%) with FAV versus 72% (95% CI 61–82%) with expectant fetal management. In the analysis, expectant management (not performing FAV) was favored if the risk of fetal demise exceeded 12% or the probability of biventricular circulation fell below 26%. For the Boston group, FAV remained favored over the plausible recent range of technical success, but this may not apply to all programs with higher fetal mortality rates or a lower biventricular circulation proportion.

It is important to note that among those who have achieved biventricular circulation, the majority have required catheter-based or surgical interventions postnatally on the aortic valve, mitral valve, and aortic arch, and endocardial fibroelastosis (EFE) resection [51,52,53]. Although initial reports on postnatal outcomes are promising, further longitudinal follow-up is essential for the biventricular circulation cohort to assess long-term survival. Left ventricular diastolic dysfunction is common in this population, raising concerns for the development of restrictive left ventricular physiology, left atrial enlargement, and pulmonary hypertension, necessitating careful monitoring.

## 3. Atrial Septal Intervention for Hypoplastic Left Heart Syndrome (Hlhs) with Highly Restrictive or Intact Atrial Septum (R/Ias)

**Figure 3:**
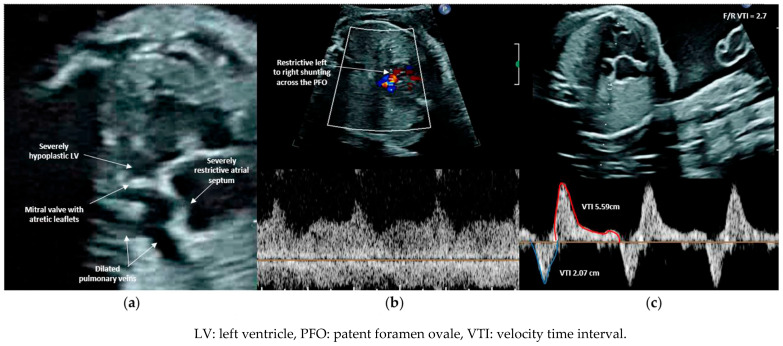
(**a**) Four chamber view demonstrating nearly intact atrial septum and dilated pulmonary veins. The left ventricle is severely hypoplastic, non-apex forming with endocardial fibroelastosis. (**b**) Restrictive continuous left to right shunting across the patent foramen ovale with a mean gradient of ~4 mmHg. (**c**) Forward to reversed velocity-time integral of <3 mm, suggesting severe atrial level obstruction.

HLHS with restrictive or intact atrial septum (R/IAS) represents a variant of HLHS, constituting approximately 6% of all HLHS cases, characterized by severe restriction or absence of flow across the foramen ovale (Figure 3). Severely restrictive or intact atrial septum leads to significant damage to the pulmonary vasculature, lung tissue, and lymphatic system during fetal development due to elevated left atrial pressure in utero [54,55,56,57,58,59,60]. At birth, with a drop in pulmonary vascular resistance, there is a sudden increase in pulmonary venous return to the left atrium, resulting in a sharp rise in left atrial pressure. This leads to extensive pulmonary venous congestion, pulmonary edema, and diminished cardiac output, causing profound hemodynamic instability, hypoxia, and acidosis within the first hours of life. Postnatal management typically involves intervention to open the atrial septum and alleviate pressure on both the left atrium and lungs.

Due to high mortality associated with HLHS with R/IAS, various postnatal approaches are employed across different institutions, including open surgical procedures, percutaneous catheter-based techniques, and ex utero intrapartum procedures (EXIT) [54,55,56,57,58,60,61,62,63]. Despite successful relief of atrial obstruction through catheter-based or surgical means, mortality rates remain high, with a 1-year survival rate ranging from approximately 33% to 54%, and survival to the Fontan stage ranging from 0% to 46%, partly attributable to lung disease and associated pulmonary hypertension [54,55,56,57,58,59,60,61,62,63,64,65].

In contrast to fetal aortic stenosis, where there is a progression of cardiac disease in utero, the rationale for FCI in HLHS with R/IAS is aimed at improving prenatal blood flow across the atrial septum to mitigate chronic pulmonary vascular changes, ultimately enhancing immediate postnatal hemodynamics and reducing severe neonatal distress [66,67,68,69,70,71].

### 3.1. Patient Selection

**Figure 4:**
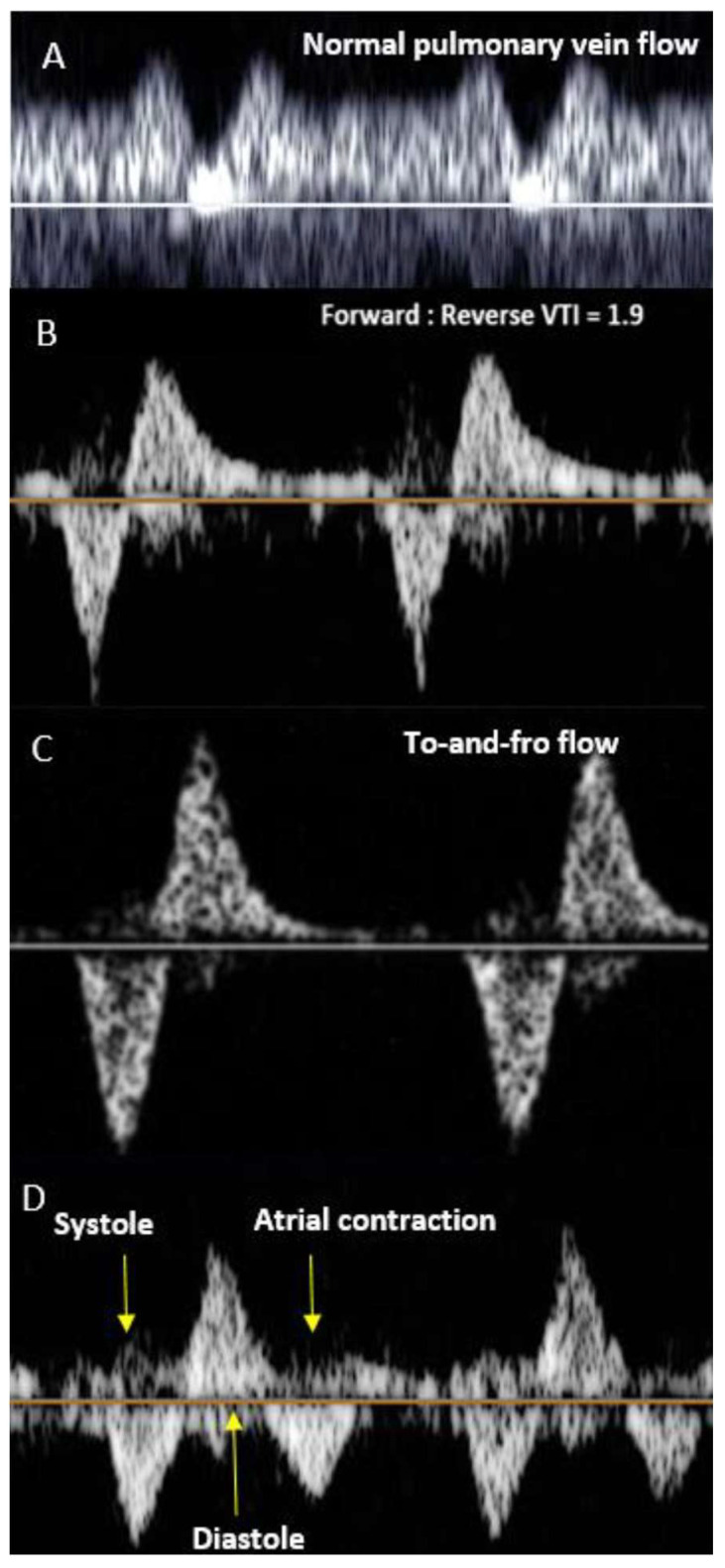
(**A**) Normal pulmonary vein flow pattern with phasic flow. No flow reversal seen in diastole. (**B**) Pulmonary vein Doppler flow pattern in HLHS with a severely restrictive atrial septum as evidenced by a forward to reversed velocity-time integral of < 3 mm. (**C**) Pulmonary vein flow pattern typically seen in the setting of an intact atrial septum, showing to-and-fro flow. (**D**) Double reversal sign seen in severely restrictive or intact atrial septum and severe mitral valve regurgitation.

Predictors from fetal echocardiograms that indicate the severity of neonatal illness in fetuses with R/IAS in left-sided obstructive disease have been previously documented [64,65]. These studies have highlighted that an abnormal pulmonary vein (Pvein) flow pattern, specifically a prograde:retrograde velocity time integral below 5, is highly sensitive (100%) and specific (94%) for predicting severe hypoxia at birth and the necessity for emergent postnatal atrial decompression. Lowering the cutoff to below 3.0 reduced false positives, achieving 100% specificity but with poorer sensitivity. The characteristics of dilated pulmonary veins and flow patterns in both normal fetuses and those with a severely restrictive or intact atrial septum are illustrated in Figure 4 . Additionally, acute maternal hyperoxygenation (AMH) has been explored as both a diagnostic tool and a provocation test to evaluate pulmonary vasodilatory capacity, thereby assessing the severity of pulmonary vasculopathy in fetuses with HLHS and R/IAS [72,73,74,75]. Szwast et al. reported that when using a cutoff value of <10% pulmonary vascular reactivity, AMH testing demonstrated a sensitivity of 100% (0.46–1.0), specificity of 94% (0.78–0.99) for determining the need for an immediate intervention at birth [72]. Mardy et al. later suggested that the Pvein VTI ratio change may more accurately identify fetuses requiring emergent atrial septal intervention than changes in the pulmonary artery pulsatility index, with less interobserver variability [75]. Due to the high specificity of the Pvein Doppler pattern in predicting the immediate need for postnatal atrial septal intervention, forward:reverse VTI < 3 has become the primary selection criterion for FCI. FCI is typically offered between 24 and 34 weeks gestational age to fetuses meeting the inclusion criteria.

### 3.2. Procedural Technique

**Figure 5:**
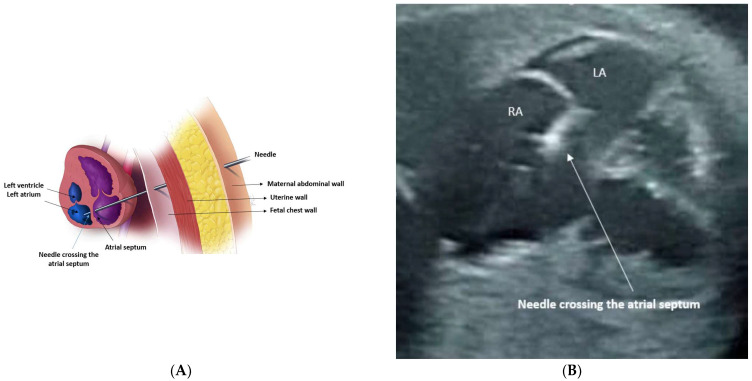
(**A**) Illustration demonstrates the needle crossing through the maternal abdominal wall, uterine wall, fetal chest into the right atrium, then through the atrial septum into the left atrium. (**B**) Under ultrasound guidance, the image demonstrates the needle crossing through the fetal chest into right atrium, then through the atrial septum into the left atrium.

**Figure 6:**
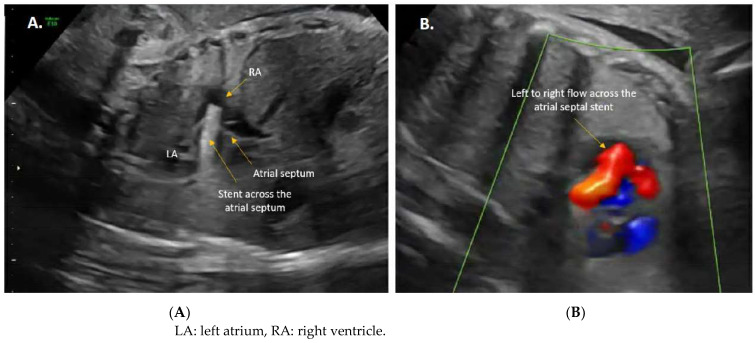
(**A**) 2D image on the left shows the atrial septal stent after it was deployed across the atrial septum. (**B**) Color image on the right shows unrestrictive, left to right flow across the atrial septal stent.

Similar to the FAV, FCI for R/IAS in the context of HLHS is also typically performed percutaneously under continuous ultrasound guidance [66,67,68,69,70,71]. It also involves epidural anesthesia rather than general anesthesia (GA), administered under conscious sedation to mitigate the risks associated with GA and potential fetal myocardial depression [49]. Once the fetus is positioned appropriately, a combination of medications is administered to the fetus to alleviate pain during the procedure and minimize movement, ensuring safety. Subsequently, an 18- or 19-gauge needle is introduced percutaneously into the fetal chest through the atrial wall (either right or left depending on fetal positioning), with the needle tip directed toward the atrial septum. Once positioned correctly, the atrial septum is punctured, followed by the introduction of a wire over the needle, and then the exchange of the needle for a balloon angioplasty catheter (Figure 5). The balloon is then inflated across the atrial septum, and in the majority of cases, a stent is placed to prevent closure of the newly created atrial–septal communication (Figure 6). The utilization of a laser to create an interatrial communication in utero was previously described by Quintero and Belfort et al. [71,76]. The authors noted that this approach offers the advantage of producing an interatrial communication unaffected by tissue recoil and is not constrained by the size limitations of the balloon or needle used during the procedure.

### 3.3. Procedural Outcomes

Regarding procedural outcomes, although the technical success of in utero intervention has been reported, fetal complications are common, including fetal demise as well as pericardial effusion and bradycardia necessitating prompt management and resuscitation in the operating room [66,67,68,69,70]. Marshall et al. reported among seven fetuses with either restrictive or intact atrial septum who underwent in utero fetal balloon atrial septoplasty between 26 and 34 weeks of gestation, in six the atrial septum was successfully perforated, with balloon dilation resulting in a small but persistent interatrial communication. However, only 57% (4/7) of cases achieved an interatrial communication >2 mm in diameter. One fetus died within four hours of the procedure due to a large right hemothorax and a small hemopericardium [66]. From the Boston cohort study that summarized 21 procedures performed over 7 years, despite a high rate of technical success (19/21 or 90%), the procedure itself was reported to be associated with fetal demise in two cases. Both of these fetuses had a moderate or large hemopericardium. Additionally, the authors reported bradycardia and pericardial effusion in seven cases (37%) [67]. Kalish et al. in 2014 presented their experience of atrial septal stent placement in nine fetuses with HLHS-IAS, with approximately 55% procedural success [69]. Multiple fetal complications were reported, including stent embolization, hemopericardium, bradycardia, heart block, and self-resolved ventricular fibrillation, with no maternal complications. IFCIR published its first report in 2015, documenting 37 fetuses with HLHS-IAS who underwent atrial septoplasty with or without stent placement. Technical success was achieved in 65% of cases, with periprocedural death reported in 5% [27]. Of the 37 cases, bradycardia requiring treatment in 10 cases and hemopericardium requiring drainage in 16 cases were reported. Subsequent updates from IFCIR in 2017 reported on 47 fetuses with HLHS-IAS undergoing atrial septoplasty (n = 27) or stent placement (n = 20), with technical success reported in 77% of cases, with 65% success in atrial stent placement [61]. However, fetal complications were common, and procedure-related fetal demise occurred in 13% of cases. The most common complications remained pericardial effusion in 24 cases, followed by bradycardia. In the largest single center experience, Schidlow et al. reported a series of 21 fetuses with HLHS-IAS undergoing atrial septoplasty, with 9% experiencing interventional fetal loss related to hemopericardium. Of 21, 13 cases resulted in an atrial communication of 2.5 mm or larger, but notably many of these communications became smaller in late gestation [74].

### 3.4. Postnatal Outcomes

As mentioned above, the primary aim of FCI is to alleviate left atrial hypertension by decompressing the left atrium and prevent structural pulmonary vascular changes, ultimately aiming for improved postnatal stability and outcomes. However, to date, no survival advantage has been established regarding the optimal timing of the procedure and the technique to achieve a persistent patent atrial-level communication after septal intervention. Marshall et al. in 2004 documented seven fetuses with either a restrictive or intact atrial septum who underwent in utero fetal balloon atrial septoplasty, with one fetal demise within four hours of the procedure and four neonatal deaths, resulting in a combined fetal and neonatal mortality rate of 71.4% (5/7) [66]. In the 2008 report, the same group provided updated data on the 19 liveborn children who survived fetal atrial septoplasty for HLHS-R/IAS. Of the 19 liveborn, 63% (12/19) required urgent atrial septal intervention, while the remaining seven neonates were stable and only required medical management prior to routine stage 1 surgery [68]. All 19 neonates underwent a stage 1 procedure with overall surgical survival of 58% (11/19). Overall survival to discharge, including fetal losses (3/21) related to the intervention, was 52% (11/21). Surgical survival was 42% (5/12) among those who underwent urgent left atrial decompression at birth. 

In 2014, Kalish et al. presented their experience of atrial septal stent placement in nine fetuses with HLHS-IAS, reporting one fetal demise [69]. Of the remaining eight fetuses that survived to delivery after undergoing atrial septal stent placement, four died in the neonatal period, resulting in a combined fetal and neonatal mortality rate of 55% (5/9). In 2015, the initial report from the IFCIR reported that survival to discharge for live-born fetuses with atrial restriction was comparable between those undergoing technically successful versus unsuccessful FCI (63.6% vs. 46.7%, respectively), although the authors noted non-uniformity in diagnostic criteria [27]. In the most recent update from the IFCIR in 2017, there was a trend indicating that stents performed better than septoplasties in maintaining a nonrestrictive atrial communication at delivery following procedural success (9/12 = 75% versus 9/23 = 39%, Fisher exact P = 0.075), with discharge survival reported at 58% (7/12) for successful stent placements [61]. However, overall discharge survival remained low with no significant difference observed between groups, either overall (34% FCI versus 36% no FCI) or with procedural success (44% successful FCI versus 33% unsuccessful or no FCI).

Although no survival benefit of FCI has been demonstrated in HLHS with R/IAS, there is an association with more stability at birth. In the most recent update from the IFCIR in 2017, among those undergoing FCI the rate of Cesarean section delivery was lower compared to those without FCI (57% vs. 86%, p = 0.049) [61]. Additionally, the proportion of those requiring Immediate Postnatal Access to Cardiac Therapy (IMPACT) or ex utero intrapartum treatment (EXIT) strategies was lower in the FCI group compared to the non-FCI group (13% vs. 67%, p < 0.01), as was the proportion needing neonatal resuscitation (33% vs. 65%, p = 0.046). Moreover, the proportion of newborns with an unrestrictive atrial septum was higher in the FCI group compared to the non-FCI group (50% vs. 22%, p = 0.03). Although newborns with FCI were generally more stable at delivery than those not undergoing FCI, the difference was not statistically significant (44% vs. 33%, p = 0.44), with the authors noting the lack of uniformity in diagnostic criteria.

## 4. Pulmonary Valvuloplasty for Pulmonary Atresia or Severe Pulmonary Stenosis with Intact Ventricular Septum (Pa/Ivs)

Pulmonary atresia or severe pulmonary stenosis with an intact ventricular septum (PA/IVS) represents a complex congenital heart defect (CHD) characterized by highly variable degrees of right heart hypoplasia, resulting in a wide spectrum of postnatal outcomes, which can range from achieving biventricular circulation following initial pulmonary valve perforation and balloon dilation to requiring single ventricle palliation or transplant in the most severe cases [77,78,79,80,81,82,83]. Coronary artery anomalies are also reported in this complex CHD and are known to complicate the postnatal course. Postnatal series have demonstrated that coronary anatomic features and RV and TV sizes are the most important determinants of univentricular versus biventricular outcomes and cardiac death [81,82,84].

Similar to fetal aortic stenosis, the increased right ventricular (RV) afterload associated with severe pulmonary stenosis or atresia is thought to trigger progressive RV hypertrophy and reduced RV compliance. Over time, this, coupled with decreased flow across the right heart, contributes to RV growth failure. PA-IVS is rare, constituting approximately 1–3% of all congenital heart defects observed in pediatric cases. However, its prevalence during fetal development is somewhat higher, with estimates nearing 5% of CHD cases [85]. The severity of this condition, the extent of right ventricular (RV) hypoplasia, and its hemodynamic disruptions primarily depend on the timing of the onset of the anatomical defect. Tricuspid regurgitation (TR) is frequently observed in fetuses diagnosed with critical PS/PA-IVS [85,86,87,88,89,90]. TR is usually severe and constitutes one of the main hemodynamic disturbances, making it unique among right heart obstructive diseases [88]. However, in the absence of high-grade TR, this lesion leads to a hypoplastic RV, which may preclude a biventricular circulation after birth [79,80,90].

Apart from TR, a mid- to late-gestation fetal tricuspid valve (TV) z-score, rate of growth, and RV size and compliance predict the postnatal outcome in PA/IVS [79,80,83,85,86,87,88,89,90]. Fetal pulmonary valvuloplasty (FPV) has been proposed in order to alter the natural progress of right-sided heart disease or to prevent RV growth failure, with an ultimate goal of preserving the tripartite RV morphology and maintaining biventricular circulation after birth [27,87,91,92,93,94,95,96].

Prenatal treatment may be a unique opportunity to promote ventricular growth better than postnatal treatment.

### 4.1. Patient Selection

Due to the absence of well-established selection criteria due to a lack of physiological markers indicating right heart adequacy, current practices rely on certain indicators to determine candidacy for FCI. TV z-score and qualitative assessment of RV size are often used to guide decision-making regarding FCI eligibility, as well as other algorithms predicting the likelihood of a single ventricle or “one and a half” palliation [27,87,91,92,93,94,95,96].

Currently, fetal pulmonary valvuloplasty is considered for fetal PA/IVS in patients with a membranous (rather than muscular) pulmonary atresia or severe pulmonary stenosis, with identifiable pulmonary valve (PV) leaflets or a membrane, or an intact or highly restrictive ventricular septum. A tricuspid valve annulus z score falling between −2.5 to −4 for gestational age may be used as an inclusion criterion, but it is not universally used and RV size should be no more than moderate RV hypoplasia. Fetuses with TV z-score above −2.5 are considered mild cases and are likely to achieve a biventricular circulation; therefore, FPV would not meaningfully change the clinical course. Although these criteria guide decision-making regarding FCI eligibility, it is also important to note that fetal pulmonary valvuloplasty may not be a feasible option for fetuses with muscular pulmonary atresia.

### 4.2. Procedural Technique

Fetal pulmonary balloon valvuloplasty is typically performed at 21 to 32 weeks of gestation [92,95,96]. Under the guidance of ultrasound, manual manipulation may be conducted as necessary to optimize the fetal position. Historically, if the optimal position could not be achieved or imaging was inadequate, a limited maternal laparotomy without uterine exteriorization or incision was performed, although recent reports do not describe laparotomy use [95,96]. Access to the RV outflow tract is attained through direct puncture, utilizing a 16, 18, or 19-gauge ultra-thin-walled cannula [92,95]. The trajectory of access is either through a subcostal approach on the fetal chest or through an intercostal space adjacent to the sternum. Once the cannula tip is positioned in the RV outflow tract, the atretic or stenotic PV is perforated either with the stylet of the cannula or with an ultrasharp, 22-gauge Chiba needle introduced through the cannula. A floppy-tipped guidewire is carefully positioned in either a branch pulmonary artery or the ductus arteriosus, followed by inflation of a coronary angioplasty balloon across the valve. Upon completion of the procedure, the wire, balloon, and cannula are removed after the final deflation of the balloon.

### 4.3. Procedural Outcomes

Technical and physiological success of FPV in promoting the achievement of biventricular circulation after birth in PAIVS/PS has been reported [27,87,91,92,95,96]. In the initial series from Boston group, 10 fetuses underwent attempted balloon dilation of the PV in utero [92]. The first four procedures were technically unsuccessful, and the most recent six were technically successful, suggesting the significant learning curve associated with the procedure. Fetal hemodynamic instability (bradycardia and left ventricular dysfunction) complicated all six cases (four unsuccessful and two successful) but resolved with treatment. One pregnancy was terminated after an unsuccessful intervention. The initial IFCIR report included 16 cases of pulmonary valvuloplasty [27]. In this multicenter study, of the 30 fetuses considered for FPV, 16 underwent FCI, and 8 met institutional criteria but did not undergo intervention. Of the 16, 11 or 69% were technically successful, 3 were technically unsuccessful, and for 2 cases, success was not described. Technical success was defined as the perforation and/or passage and dilation of the pulmonary valve. Of the 11 technically successful cases, there were 3 periprocedural and 1 late fetal demise (4/11 or 36%). Of the two technically unsuccessful cases, one had a periprocedural death (1/2 or 50%). Complications were not insignificant, including hemopericardium requiring drainage in nine (56%) and bradycardia requiring treatment in seven (44%). There were no maternal complications. Of note, in this multicenter study, the inclusion criteria were highly variable.

Tulzer et al., in 2018, described 23 cases of PA/IVS (15) and severe pulmonary stenosis (8) [95]. Thirty-five pulmonary valvuloplasties were performed on 23 fetuses. No fetal deaths occurred in this cohort. The outcomes of this study were better than the prior studies for both technical success and fetal complications. A procedure was considered successful if the pulmonary valve was perforated and/or passed and dilated with a balloon catheter. A partially successful procedure was defined as one in which the pulmonary valve was perforated and passed, but the valve was not fully dilated with the balloon. Seventeen patients had a technically successful procedure, and four had a partially successful procedure, resulting in an overall success rate of 91.3%. Pericardial effusion necessitating treatment occurred in 4/35 (11.4%) and persistent bradycardia in 11/35 (31.4%) procedures.

In their subsequent IFCIR report, the authors broadened the scope of the initial report and queried the IFCIR for PAIVS/PS cases from January 2001 to April 2018 [96]. This report from the IFCIR is still the largest published cohort. Of the 84 maternal/fetal dyads in this registry, 58 underwent pulmonary valvuloplasty at a median gestational age of 26.1 (21.9–31.0) weeks. Characteristics of fetuses undergoing FCI varied in terms of TV size, TR, and PV patency. The procedure was technically successful in 41 cases (41/58 or 71%), not successful in 15 cases (15/58 or 26%), and success was not reported in 2 cases. There were fetal complications in 55% of cases, including seven periprocedural (four at <48 h post-operatively and three during the procedure) deaths and two delayed fetal losses (15.5%). Both of the late fetal deaths had developed a circular shunt from significant pulmonary insufficiency after FCI and were thought to expire due to associated unfavorable hemodynamics. There were no maternal complications reported. One or more fetal complications occurred in more than half (32/58, 55%) of the cases, the most common being a pericardial effusion requiring drainage (28/58, 48%) or bradycardia requiring treatment (21/58, 36%). There were three cases of a pleural effusion or hemothorax. Overall, there were notable rates of technically unsuccessful procedures and complications associated with the procedure, including fetal loss as summarized above.

### 4.4. Postnatal Outcomes

In the Boston cohort published in 2012, nine fetuses progressed to term and underwent neonatal interventions, including balloon pulmonary valvuloplasty in six cases and surgical procedures in eight cases, and were alive at follow-up times ranging from 0.5 to 5.8 years of age [79]. Four of the six patients (67%) who were live born after successful prenatal interventions achieved biventricular circulation (vs. 0 biventricular circulation in the technically unsuccessful group) whereas the fifth had a very small RV with an RV-dependent coronary circulation and the sixth had an intermediate palliative circulation with a RV size and function favorable for biventricular repair. Overall, also including fetal procedural demise, the percent alive with biventricular circulation was 40%. These outcomes were compared with 15 control fetuses with PA/IVS. Of the 15 control fetuses, 6 had biventricular (40%) and 9 had univentricular outcomes (60%), suggesting that their overall outcomes were not greatly different. In the technically successful group, the authors reported the maintenance of valvar patency throughout gestation and continued growth of the right heart structures. Because of the small numbers and limited follow-up data on fetuses who underwent unsuccessful procedures, the growth trajectory of the right heart structures were unable to be compared between fetuses who underwent successful versus unsuccessful interventions.

In Tulzer et al.’s cohort in 2018, the biventricular outcome after successful FPV was comparable to the Boston study [95]. Fetuses with unsuccessful intervention (n  =  2) became univentricular; all others had either a biventricular (n  =  15) (15/23 or 65%), one-and-a-half ventricular (n  =  3) or still undetermined (n  =  3) outcome. The authors reported that immediately after successful intervention, growth of the right-sided structures improved and the TR velocity decreased significantly. Of note, the mean fetal TV annulus Z-score in multiple cohorts was between −1 and −2, suggesting that the intervention cases included those that may have been predicted to undergo biventricular repair without intervention, complicating the interpretation of the results.

The initial IFCIR report in 2015 documented 16 cases of pulmonary valvuloplasty, as described previously. In this cohort, there was no significant difference in biventricular outcomes between those who underwent fetal pulmonary valvuloplasty (FPV) compared to those who did not undergo fetal cardiac intervention (FCI) [27]. Specifically, 42.9% (6 out of 14) of patients who received FCI and 37.5% (three out of eight) of those who did not undergo intervention were discharged with biventricular circulation. Among the technically successful cases, 5 out of 11, or 45%, achieved biventricular circulation, compared to 33% in the technically unsuccessful cases, where only one out of three cases achieved biventricular circulation.

In the subsequent IFCIR report, of the 84 maternal/fetal dyads included in the registry, 58 underwent pulmonary valvuloplasty as detailed above with a procedural success of 71% (41/58) [96]. Of the 41 technically successful FPV, 35 or 85% (35/41) were alive at birth as compared to 73% (11/15) in the technically unsuccessful group. There were 12 cases that did not undergo FPV and they all were alive at birth. Overall, there was a higher percentage having a biventricular circulation following successful FCI. Of those who underwent FPV and were alive at birth, a biventricular outcome was achieved in 77% (27/35), and 8% had a 1 ½ ventricular circulation (vs. 36% with a biventricular outcome (4/11) in the technically unsuccessful cohort). There were no neonates who underwent successful FCI with single ventricle physiology at the last assessment, although the ventricular status was unknown for one neonate (vs. 18% with a single ventricle outcome in the technically unsuccessful group). The rate of biventricular outcomes among cases that did not undergo FPV was comparable to those who had unsuccessful FPV (4/12, or 33%). Again, in this cohort, the mean tricuspid valve z-score was between −1 and −2, suggesting some fetuses may have not been at risk for single ventricle physiology.

Fetuses who underwent technically successful fetal pulmonary valvuloplasty maintained their growth trajectory, with stable z-scores throughout gestation (the absolute measurement of the TV increased by 0.32 (±0.17) mm/week from intervention to birth). In contrast, unsuccessful fetal cardiac intervention (FCI) cases exhibited right ventricular (RV) growth failure, as evidenced by decreasing z-scores over time.

Different from the other two CHDs discussed above, strategies aimed at postnatal management, focusing on the rehabilitation of the right heart by alleviating RV outflow tract obstruction and enhancing RV filling to achieve either a 1 ½ or biventricular circulation, have demonstrated success in certain instances, although the inclusion criteria are still undefined and varied, making interpretation of the results challenging,

## 5. Discussion Regarding Maternal Risks

The main goal of catheter-based FCI is to improve fetal hemodynamics and ultimately improve the postnatal outcome. However, the safety of the mother is of utmost importance; hence, maternal well-being during and after the procedure should also be closely monitored by the maternal anesthesia and maternal–fetal medicine teams, respectively. Catheter-based procedures require crossing the maternal abdomen and uterus with a needle, through which a catheter, wires, and devices are manipulated. These interventions have the potential to cause maternal morbidity and potentially, mortality. Thankfully, maternal complications are rare, and the most commonly reported maternal complications to date include nausea, abdominal pain, and bruising. So far, no maternal mortality and/or intensive care admission after FCI has been reported, and the only serious complications have been 1 placental rupture and 1 postpartum hemorrhage [96,97]. Indeed, Rebizant and colleagues examined the impact of FCI on maternal well-being, pregnancy progression, and childbirth [5]. Notably, there were no instances of procedure-related preterm premature rupture of membranes (pPROM), chorioamnionitis, wound infection, or complications associated with anesthesia. Among the patients, six out of nine (67%) showed resolution of polyhydramnios associated with cardiac failure following FCI, suggesting a potential role in preventing pregnancy complications related to polyhydramnios, such as cervical shortening, preterm delivery, and mirror syndrome. This finding highlights the broader potential benefits of FCI beyond cardiac outcomes, encompassing the management and prevention of various pregnancy-related complications.

## 6. Conclusions

Catheter-based FCI offers the ability to change the fetal hemodynamics and disease course (Table 1). FAV is offered to cases with critical aortic stenosis to stop progression to HLHS by relieving the left ventricular outflow tract obstruction and improving blood flow through the left heart. Early findings suggest that FAV may enhance the likelihood of achieving a biventricular repair, but this intervention carries the risk of fetal mortality, and its impact on overall mortality rates and outcomes remains uncertain. Despite a successful in utero intervention, there is still a significant disease burden postnatally that needs to be addressed via aortic and/or mitral valve intervention (catheter based vs. surgical) or EFE stripping to improve diastolic dysfunction. Therefore, the potential benefits of FAV must be carefully weighed against the known risk of fetal demise, as well as the possibilities of a technically unsuccessful procedure or postnatal SV circulation despite successful FAV. Intervention for pulmonary valve disease remains understudied, likely due to highly variable inclusion criteria and a lack of models that predict right ventricular dependent coronary arteries, which confer the highest risk of morbidity and mortality. Strategies aimed at postnatal management, focusing on the rehabilitation of the right heart by alleviating RV outflow tract obstruction and enhancing RV filling to achieve either a 1 ½ or biventricular circulation, have demonstrated success in certain cases of PA/IVS. Therefore, the survival benefit of FPV is still questionable. Unlike fetal aortic or pulmonary stenosis, where there is a progression of cardiac disease in utero, the justification for FCI in HLHS with R/IAS focuses on enhancing prenatal blood flow across the atrial septum to alleviate chronic pulmonary vascular alterations. This approach aims to improve the immediate postnatal hemodynamics and decrease severe neonatal distress. Although earlier studies reported more neonatal stability at birth, no survival benefit of FCI has been demonstrated yet in HLHS with R/IAS, although the samples are small.

Finally, fetal cardiac interventions represent a significant advancement in prenatal care, offering potential life-saving treatments for congenital heart defects as detailed in this report. However, it is also important to mention that these procedures come with ethical considerations that must be navigated with care. Balancing the potential benefits against the risks, ensuring informed consent, considering the short and not-well-known long-term outcomes, and resource implications are crucial components of the ethical landscape surrounding these interventions. Additionally, financial costs can vary significantly based on factors such as location, the specific medical center, and the complexity of the case. Insurance coverage varies, and many families may need to seek additional financial assistance to manage these expenses. This should be discussed with the family during the fetal consultation session before proceeding with the fetal intervention.

## 7. Future Directions

In summary, catheter-based FCI offers the ability to change the fetal hemodynamics and the disease course. However, the survival benefits are questionable and future work should focus on identifying the ideal candidates, optimizing the techniques, and minimizing fetal periprocedural mortality by refining the inclusion and exclusion criteria based on the patient characteristics. Addressing the current limitations such as the absence of widely established inclusion criteria, restricted postnatal outcome data due to the limited number of PA/IVS cases undergoing FCI, and the lack of long-term follow-up, may be feasible through larger-scale studies and a collaborative approach.

## Figures and Tables

**Table 1:** Summary of Catheter-based Fetal Cardiac Intervention Outcomes.

Catheter-Based FCI	Goal	Maternal Risk	Risk of Fetal Demise	Need for Early Postnatal Intervention	Short-Term Outcomes FCI Vs. No or Unsuccessful FCI	Long-Term Outcomes
**FAV**	Improve likelihood of biventricular circulation	Minimal (see maternal risks)	4–32%, pooled 16%	Common *	BiV no/unsuccessful FAV: 0–41% BiV FAV all comers: 28–45%, pooled 37% BiV among TS FAV liveborn: 39–68%, pooled 52% Survival (last FU) no/unsuccessful FAV: 0–100% Survival (last FU) FAV all comers: 21–75%, pooled 73% at 1 y Survival (last FU) among TS FAV liveborn: 55–74% (no pooled est.) Summary: reasonable evidence for higher likelihood of BiV repair. Insufficient data for improved survival to date, as many survival reports are limited to those who successfully achieved BiV repair and do not account for overall survival. Expectant management (no FAV but meeting criteria) also inconsistently reported.	Unknown
**FASI**	Improve stability at birth, improve survival	Minimal (see maternal risks)	8–33% pooled 10%	Common *	Postnatal R/IAS among no/unsuccessful FASI: 41–100%, pooled 89% Postnatal R/IAS among FASI all comers: 38–50%, pooled 47% Postnatal R/IAS among TS FASI liveborn: 17–50% (no pooled est.) Survival to DC among no/unsuccessful FASI: 33–76%, pooled 57% Survival to DC among FASI all comers: 29–100%, pooled 46% Survival to DC among TS FASI liveborn: 44–60% (no pooled est.) Summary: reasonable evidence for less restriction at birth, although inclusion criteria for FASI are variable. Insufficient data for improved hospital discharge survival.	Unknown
**FPV**	Improve likelihood of biventricular circulation	Minimal (see maternal risks)	0–36%, IFCIR 16%	Common *	BiV no FPV: 0–100%, IFCIR 33% BiV all FPV comers: 31–65%, IFCIR 56% BiV among TS FPV liveborn: 70–83%, IFCIR 77% Survival to DC no FPV: IFCIR 75% Survival to DC FPV all comers: IFCIR 75% Survival to DC among TS FPV liveborn: 89% Summary: FPV seems associated with a high likelihood of BiV repair, but no standard inclusion criteria or standard controls. Many subjects in the studies seem likely to have progressed to 1.5 or BiV based on prediction algorithms without intervention. Still insufficient data if FPV better than expectant management or who optimally would benefit from intervention. Insufficient data for improved survival to date.	Unknown

BiV: biventricular; est.: estimate; FASI: fetal atrial septal intervention; FAV: fetal aortic valvuloplasty; FCI: fetal cardiac intervention; FPV: fetal pulmonary valvuloplasty; IFCIR: International Fetal Cardiac Intervention Registry; TS: technically successful. Pooled estimates from FAV and FASI from systematic reviews [98,99]. For FPV, the largest study to date is from multicenter IFCIR study, so those pooled estimates are reported [96]. * Highly variable and often incomplete how reported. The vast majority of newborns still need interventions within all three groups. Other sources for table: [2,27,40,43,48,50,70,92,100,101,102,103,104,105,106,107,108,109].

## Data Availability

No new data were created or analyzed in this study. Data sharing is not applicable to this article.
